# Supplementary material for: Comprehensive Analysis of FASN in Tumor Immune Infiltration and Prognostic Value for Immunotherapy and Promoter DNA Methylation
Source: Int J Mol Sci. 2022 Dec 9;23(24):15603. doi: 10.3390/ijms232415603 (PMC9779179; doi:10.3390/ijms232415603)
Supplement: Supplementary file 1 [file ijms-23-15603-s001.zip › ijms-2034761-supplementary/Supplementary Figure Legends.docx]

**
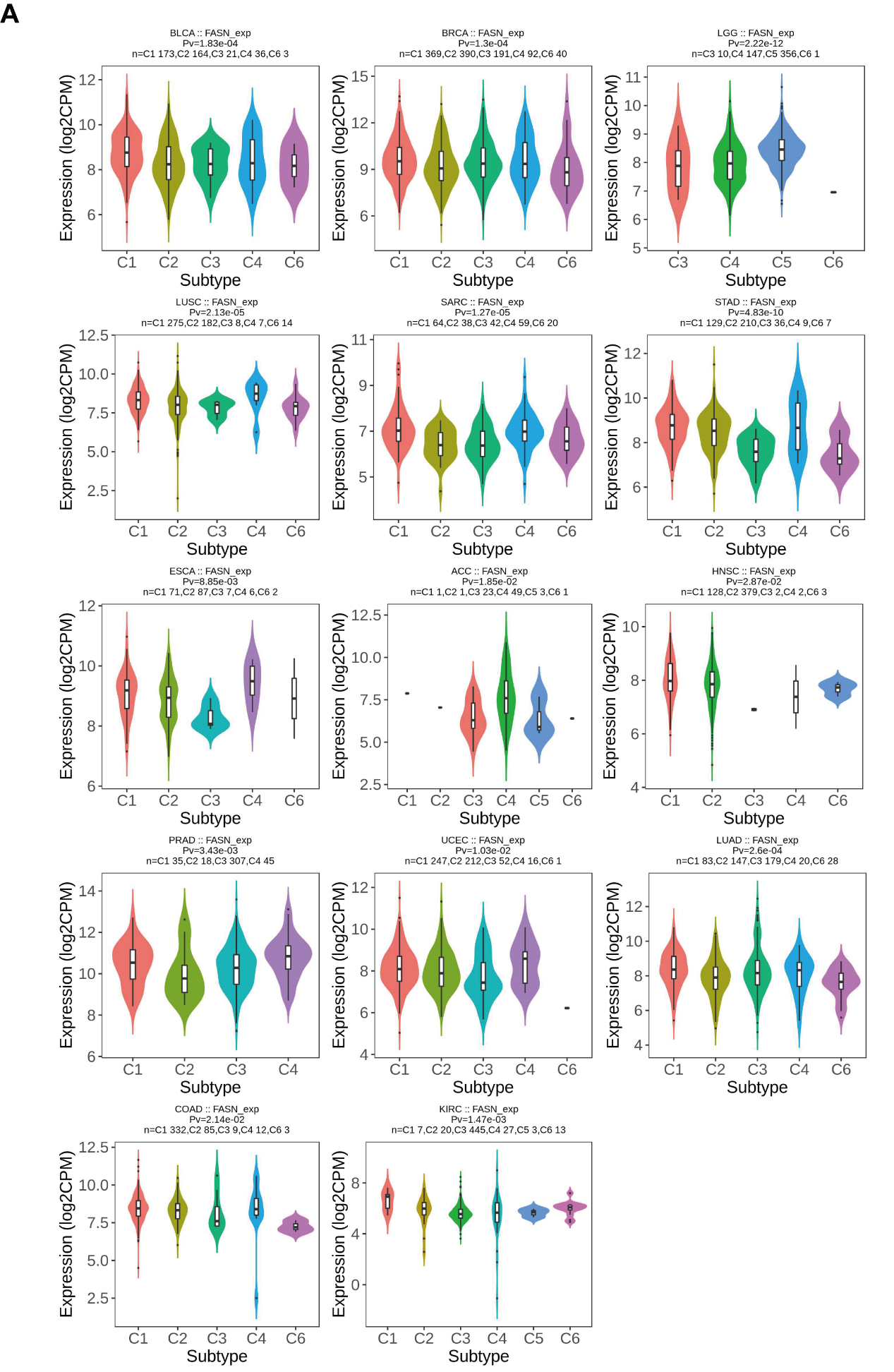
**

**Supplementary Figure S1.** FASN expression correlates with immune subtype in 14 cancer types (BLCA, BRCA, LGG, LUSC, SARC, STAD, ESCA, ACC, HNSC, PRAD, UCEC, LUAD, COAD, KIRC).

**Supplementary Figure S2.** Prognostic analysis of transcription factor CIITA and correlation of FASN expression with 60 immune checkpoint genes, 41 chemokines, and 18 chemokine receptors. (A) FASN expression and clinical data. (B) Forest plot showing the prognostic value of CIITA in pan-cancer. (C) Heat map showing the correlation of FASN expression with 60 immune checkpoint genes. (**p* < 0.05) Heat map showing the correlation of FASN expression with chemokines (D) and chemokine receptors (E) (**p* < 0.05, ***p* < 0.01, ****p* < 0.001.).


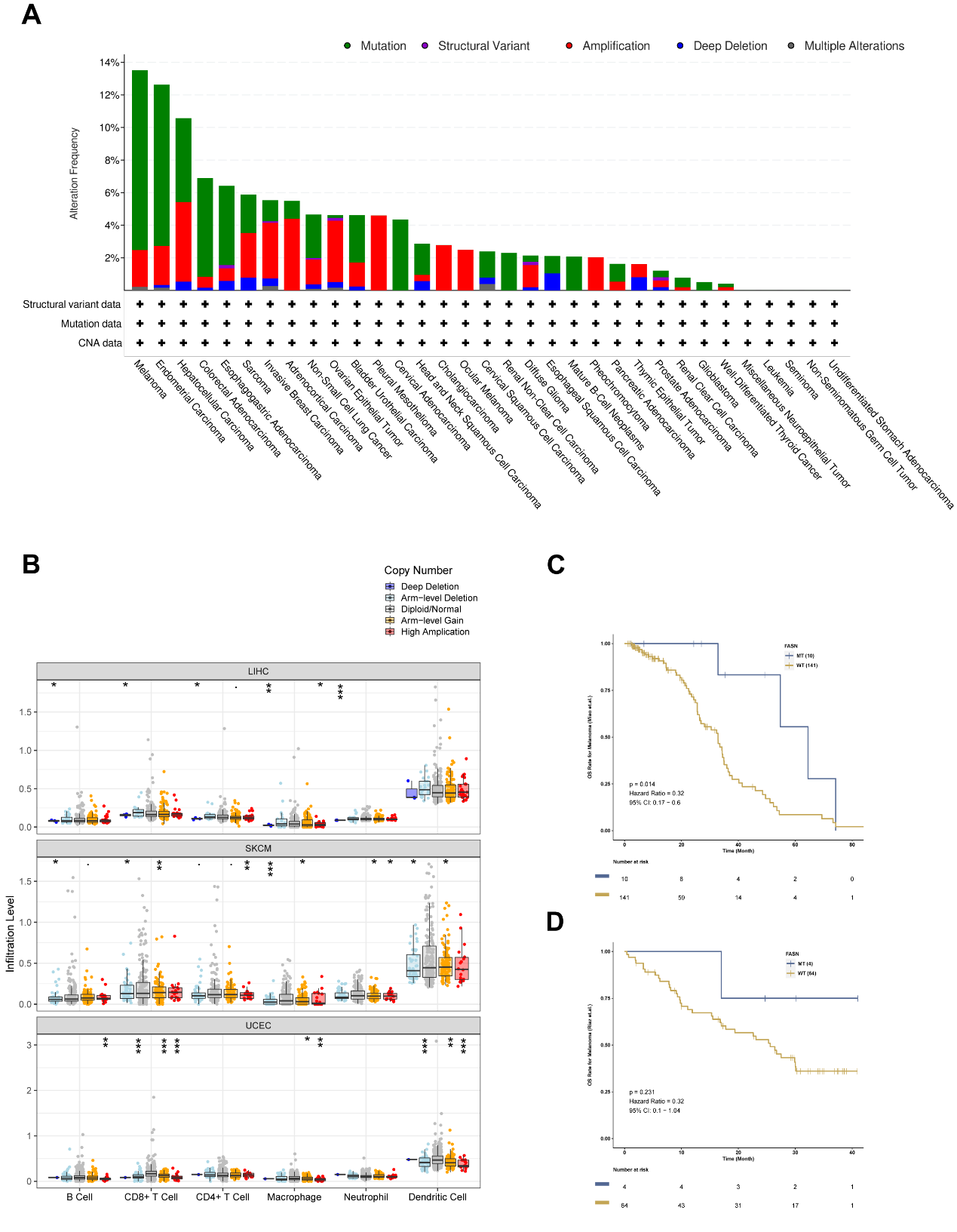


**Supplementary Figure S3.** Mutations of FASN and effect of altered somatic cell copy number of FASN on tumor infiltration. (A) Bar chart of FASN gene variants in 32 cancer studies based on TCGA PanCancer Atlas study. (B) Analysis of the effect of FASN gene variants on immune cell infiltration in the three cancer types with the most FASN variants, LIHC, SKCM, and UCEC. (C) and (D) The CAMOIP database was used to analyze the effect of FASN mutation status on the clinical prognosis of melanoma patients after immunotherapy.


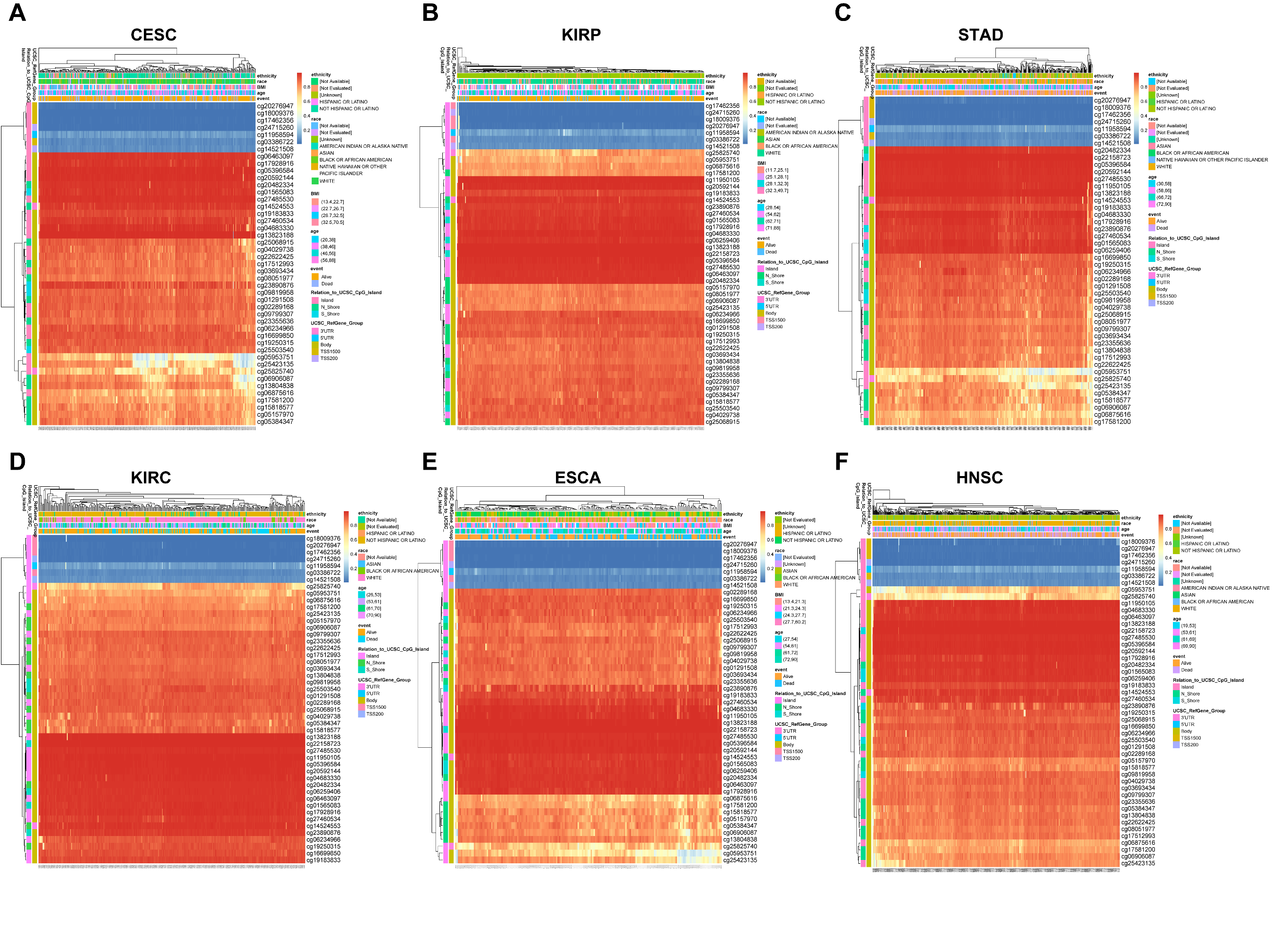


**Supplementary Figure S4.** Heatmap of DNA methylation distribution at FASN loci in 6 FASN-expressing tumors. (A) CESC (B) KIRP (C) STAD (D) KIRC (E) ESCA (F) HNSC.


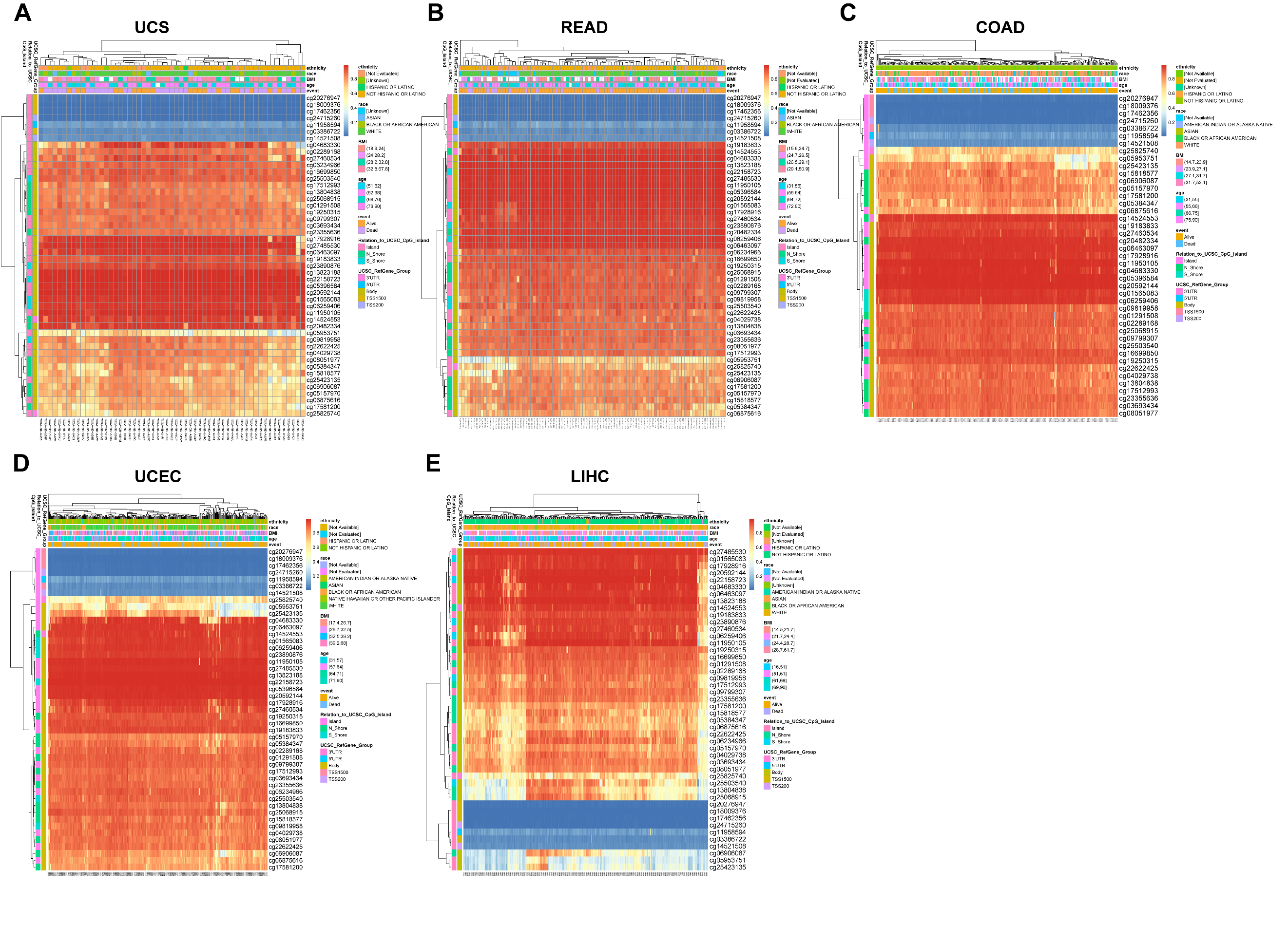


**Supplementary Figure S5.** Heatmap of DNA methylation distribution at FASN loci in five FASN-expressing tumors. (A)UCS (B) READ (C) COAD (D) UCEC (E)LIHC.


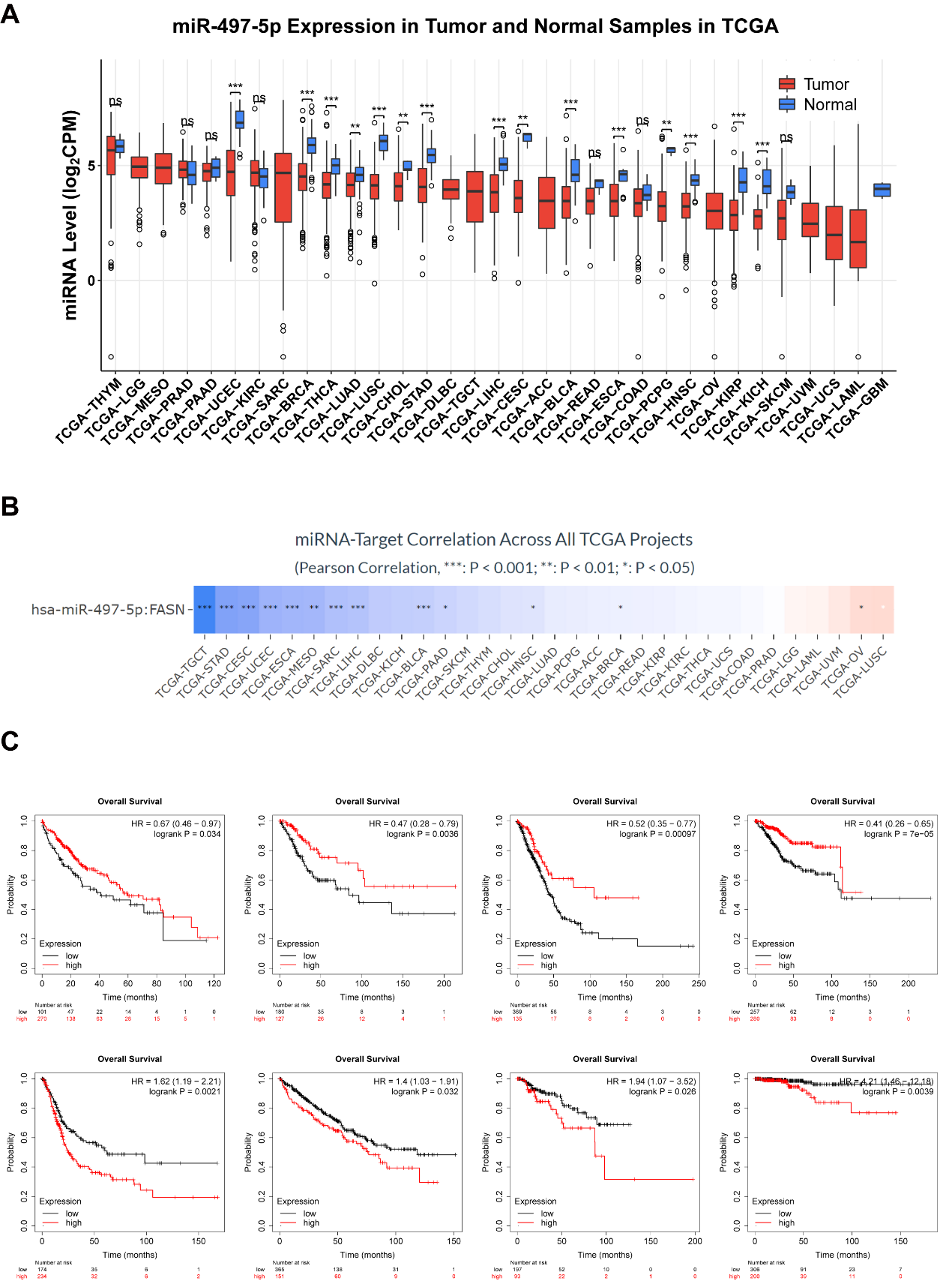


**Supplementary Figure S6.** Mir-497-5p expression in pan-cancer, correlation with FASN, and prognostic analysis. (A) mir-497-5p Expression in tumor and normal samples in TCGA (**p* < 0.05, ***p* < 0.01, ****p* < 0.001, ns: *p* > 0.05.). (B) Correlation of mir-497-5p with FASN (**p* < 0.05, ***p* < 0.01, ****p* < 0.001.). (C) The prognosis of mir-497-5p expression in pan-cancer, and the survival map and Kaplan-Meier curves with positive results are given.


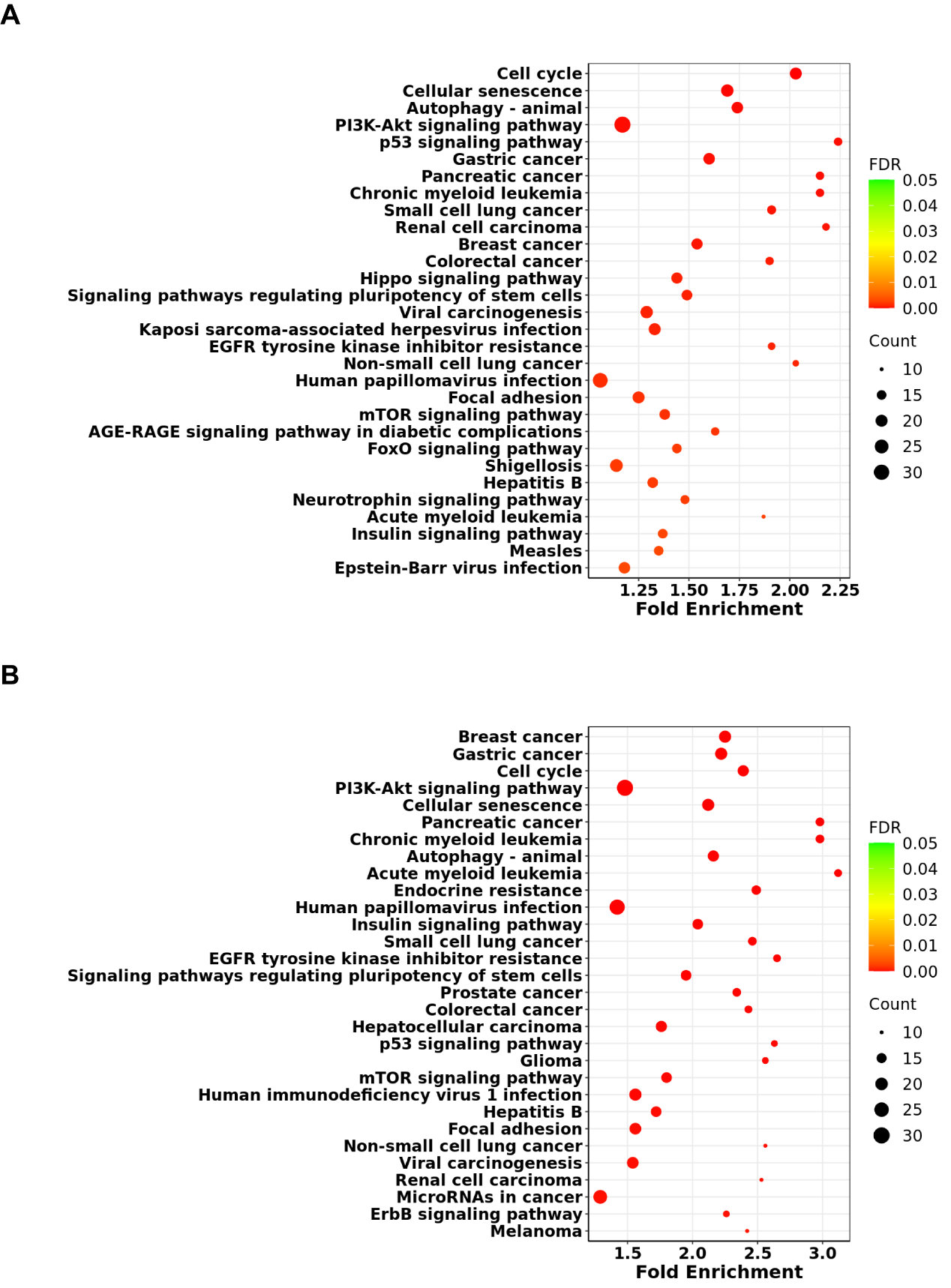


**Supplementary Figure S7.** Bubble plot of the top thirty enrichment pathways for miRNA target genes. KEGG enrichment pathways for mir-195-5p (A) and mir-497-5p (B) target genes, respectively.


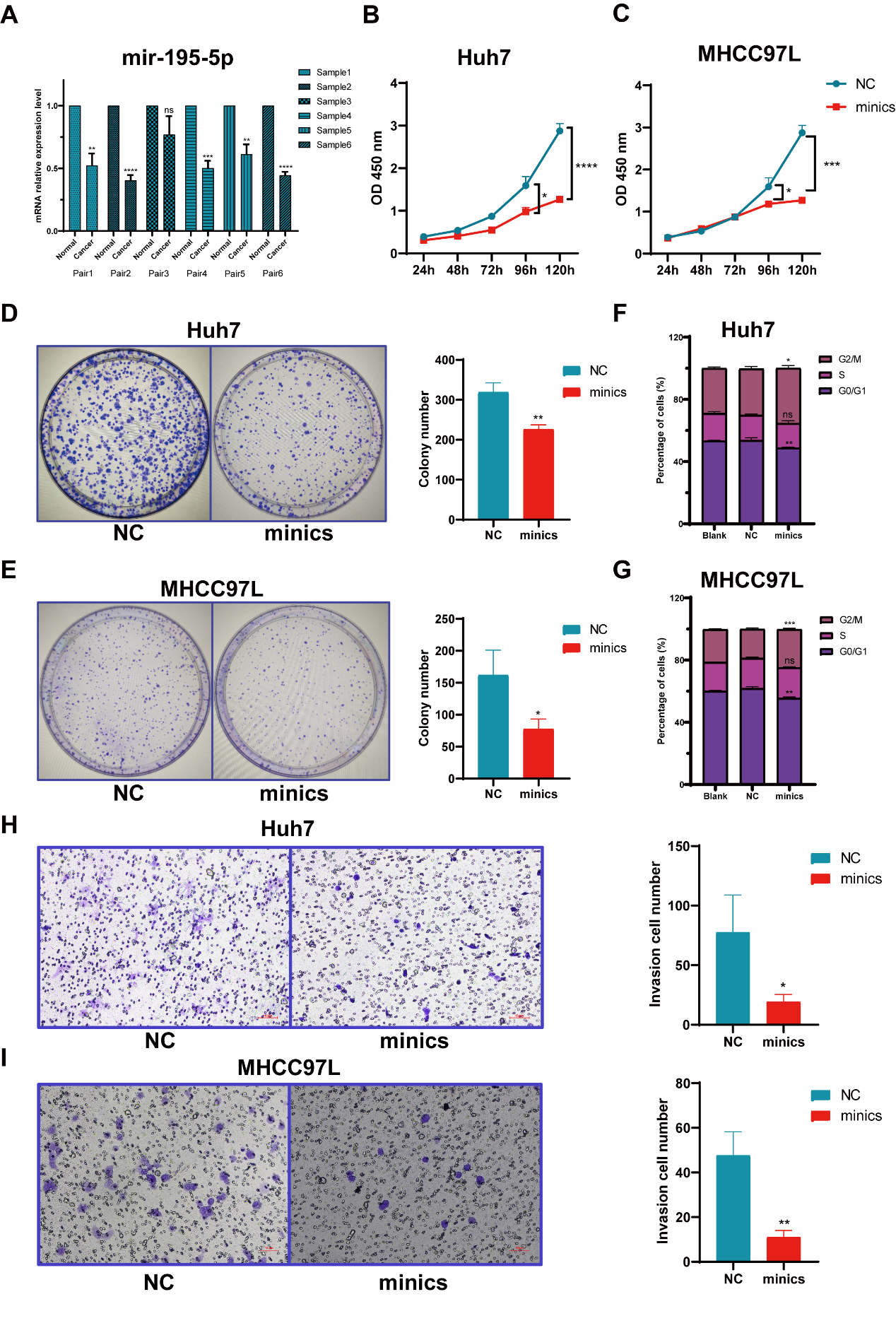


**Supplementary Figure S8.** Expression of mir-195-5p in tissue samples from hepatocellular carcinoma patients and oncogenic effects in hepatocellular carcinoma cell lines. (A) Expression of mir-195-5p relative to the corresponding paracancerous tissue in 6 tumor samples using Quantitative-PCR. The inhibitory effect of FASN on hepatocellular carcinoma cell lines was demonstrated by CCK-8 (B,C), clone formation (D,E), cell cycle (F,G), and Transwell assays (H,I), respectively (**p* < 0.05, ***p* < 0.01, ****p* < 0.001, *****p* < 0.0001.).

To prove the authenticity of our data, we have provided all the raw data in this link. ( https://www.jianguoyun.com/p/DfT4kuMQr7rnChj0v9IEIAA )
